# Bruton’s tyrosine kinase: an emerging targeted therapy in myeloid cells within the tumor microenvironment

**DOI:** 10.1007/s00262-021-02908-5

**Published:** 2021-04-05

**Authors:** Logan Good, Brooke Benner, William E. Carson

**Affiliations:** 1 Comprehensive Cancer Center, The Ohio State University, Columbus, OH USA; 2 Department of Surgery, Division of Surgical Oncology, Tzagournis Medical Research Facility, The Ohio State University, Columbus, OH USA

**Keywords:** Bruton’s tyrosine kinase, BTK, Myeloid-derived suppressor cells, Tumor-associated macrophage

## Abstract

Bruton’s tyrosine kinase (BTK) is a non-receptor kinase belonging to the Tec family of kinases. The role of BTK in B cell receptor signaling is well defined and is known to play a key role in the proliferation and survival of malignant B cells. Moreover, BTK has been found to be expressed in cells of the myeloid lineage. BTK has been shown to contribute to a variety of cellular pathways in myeloid cells including signaling in the NLRP3 inflammasome, receptor activation of nuclear factor-κβ and inflammation, chemokine receptor activation affecting migration, and phagocytosis. Myeloid cells are crucial components of the tumor microenvironment and suppressive myeloid cells contribute to cancer progression, highlighting a potential role for BTK inhibition in the treatment of malignancy. The increased interest in BTK inhibition in cancer has resulted in many preclinical studies that are testing the efficacy of using single-agent BTK inhibitors. Moreover, the ability of tumor cells to develop resistance to single-agent checkpoint inhibitors has resulted in clinical studies utilizing BTK inhibitors in combination with these agents to improve clinical responses. Furthermore, BTK regulates the immune response in microbial and viral infections through B cells and myeloid cells such as monocytes and macrophages. In this review, we describe the role that BTK plays in supporting suppressive myeloid cells, including myeloid-derived suppressor cells (MDSC) and tumor-associated macrophages (TAM), while also discussing the anticancer effects of BTK inhibition and briefly describe the role of BTK signaling and BTK inhibition in microbial and viral infections.

## Introduction

**Fig. 1:**
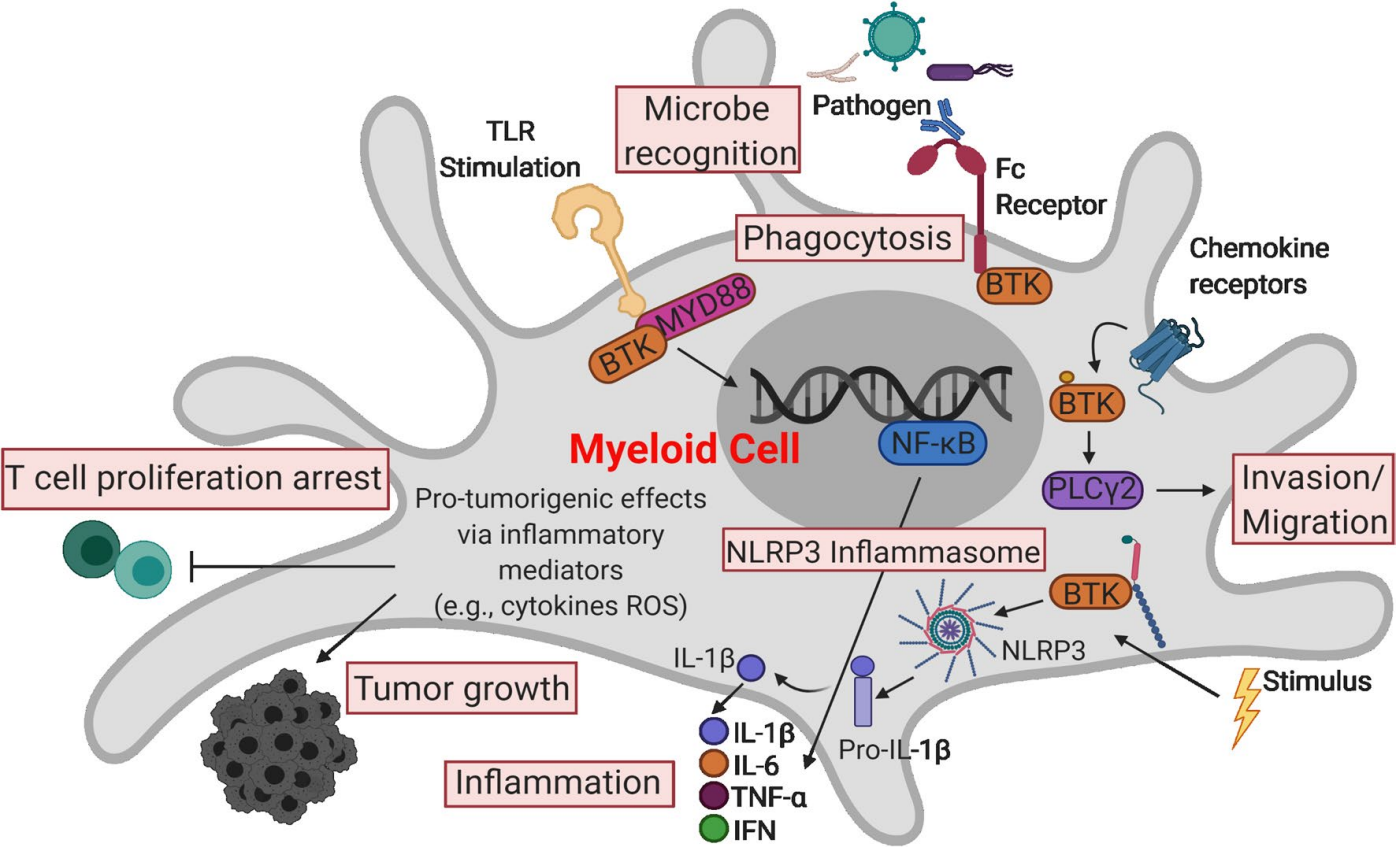
An overview of the roles of BTK in myeloid cells. The processes highlighted in red boxes indicate roles of BTK signaling in myeloid cells that have been reported in human or mice in the setting of microbial infection and cancer. Details regarding each cellular process are outlined in the text. Abbreviations: BTK, Bruton’s tyrosine kinase; TLR, Toll-like receptors; MYD88, myeloid differentiation primary response 88; PLCγ2, phospholipase C gamma 2; NF-κB, nuclear factor-kappa B; NO, nitric oxide; IL-1β, interleukin 1 beta; IL-6, interleukin 6; TNF-α, tumor necrosis factor-alpha; IFN, interferon

Bruton’s tyrosine kinase (BTK) is a non-receptor intracellular kinase that belongs to the Tec (tyrosine kinase expressed in hepatocellular carcinoma) family [1, 2]. Generally, BTK is located in a cytoplasmic position, yet it can be briefly recruited to the cell membrane via interaction with phosphatidylinositol-3,4,5-triphosphate (PIP_3_), a phospholipid effector activated by phosphatidylinositol-3 kinase (PI3K) [3]. BTK is known for its role in B-cell receptor (BCR) signaling, which is critical for normal B cell development and survival as well as its involvement in the signaling of Toll-like receptors (TLRs), chemokine receptors, and growth factor receptors. However, the expression of BTK is not restricted to B cells [2, 4–8]. Many myeloid cell lineages including monocytes, macrophages, thrombocytes, neutrophils, dendritic cells, and other cell types also express BTK, although the role that BTK plays in myeloid cell function is less defined [9, 10]. Recently, our group and others have shown that BTK is involved in the function, maturation, and trafficking of myeloid cells and plays an important role in the regulation of myeloid cell signal transduction [9–13]. BTK has an impact on a variety of myeloid cell pathways, including signaling through the NLRP3 inflammasome in neutrophils and macrophages, receptor activation of nuclear factor-κβ (NF-κB in osteoclasts, and the function and activation of myeloid-derived suppressor cells (MDSC) (Fig. 1) [14–18]. Research has shown that BTK also plays a key role in the oncogenic signaling that is critical for the survival and proliferation of several B cell malignancies [19]. BTK has been identified as a therapeutic target in several hematological malignancies, which has driven the development of small-molecule inhibitors that were efficacious in preclinical studies and have gone on to testing and approval in the clinical setting [20, 21]. Finally, it is important to note that BTK is also expressed by T cells and NK cells and is significant in their activation [22, 23]. However, the focus of this review is on the role of BTK in myeloid cells and the therapeutic implications of targeting BTK in cancer and microbial infections.

## BTK in BCR signaling and B cell malignancies and disorders

BTK is critical for B cell development and BCR signaling. The BCR signaling pathway is important in regulating the ability of B cells to survive, mature, adhere, and produce antibodies as plasma B cells [19, 24–26]. Upon BCR engagement, a signaling cascade involving Lyn, Syk, and BTK results in the activation of phospholipase Cγ2 (PLCγ2). In turn, the transcription factor NF-κB is activated resulting in the stimulation of survival and proliferative pathways [27–30]. In the absence of BTK, BCR-induced survival and proliferative signals are diminished [27–29].

BTK also contributes to tumor cell proliferation and survival in B cell leukemia and lymphoma [31]. Evidence suggests that overactive signaling supports the development of the tumor microenvironment through aberrant chemokine-controlled integrin-mediated adhesion and migration of malignant cells [26, 32]. Various small molecule inhibitors have been developed to target protein kinases in the BCR signaling cascade, and these have shown promising clinical effectiveness in multiple disease settings [2, 33]. Overall, this emphasizes the potential of targeting molecules associated with the BCR signaling pathway.

Defects in BTK have been implicated in abnormal B cell function. One major consequence of BTK impairment is the developmental delay of pro-B cells to pre-B cells and onto mature B lymphocytes. In 1993, BTK was identified as the gene defective in X-linked agammaglobulinemia (XLA), an inherited immunodeficiency disease in humans characterized by a drastic deficiency in B lymphocytes, low levels of immunoglobulin (Ig), and recurring infections [34–37]. Mutations in the pleckstrin homology (PH) domain reduce the binding activity of BTK and eliminate downstream signaling, which was identified in the murine version of BTK deficiency known as X-linked immunodeficient (XID). XID presents with a set of physiological manifestations similar to that of XLA in humans, yet is not as severe [34–38]. Both XLA in humans and XID in mice result in the depletion of B cells.

## BTK in immune regulation and function

BTK is also expressed by other immune cell populations and has been implicated in the immune regulation and function of myeloid cells. BTK has been shown to play an important role in many TLR signaling pathways. TLRs are both extracellular and intracellular pattern recognition receptors that are expressed in B cells and myeloid cells. They recognize structurally conserved molecules from bacteria and viruses [19]. Upon activation by pathogens, most TLRs recruit the adaptor myeloid differentiation primary response 88 (MyD88) which activates interleukin-1 receptor-associated kinase1 (IRAK1). This results in the activation, proliferation, antibody secretion, class switch recombination, and pro-inflammatory cytokine production in B cells [19, 39–41]. Studies investigating non-canonical pathways activated downstream of TLRs have shown that BTK can interact with the adapter molecules MyD88 and MAL downstream of TLR4, 6, 8, and 9 ultimately resulting in the production of IL-6 and TNF-α (Fig. 1) [42, 43]. Additionally, BTK is required for TLR-mediated IL-10 production in B cells and the cooperation between BCR and TLR signaling in the enhancement of IL-6 expression [38]. Kawakami et al. reported that BTK is required for IL-10 secretion by DCs and subsequent activation of STAT3 [13]. Notably, in the setting of BTK deficiency, dendritic cell cytokine production is impaired in response to viral single-stranded RNAs that activate TLR-8-mediated TNF-α and IL-6 production (*Fig. **1*) [6].

Furthermore, BTK is a contributor to chemokine receptor signaling pathways. Integrin α4β (VLA-4)-mediated adhesion of B cells to vascular cell adhesion molecule-1 and fibronectin is dependent on BTK, as are B cell responses to various chemokines like stromal cell-derived factor 1 (SDF-1) and chemokine (C-X-C motif) ligand 13 (CXCL-13) [44–46]. Chemokine receptors are G-protein-coupled receptors that are composed of seven transmembrane regions, an extracellular N-terminus contributing to ligand specificity, and intracellular hetero-trimeric G proteins comprised of α, β, and γ subunits (Gα, Gβ, and Gγ [47, 48]. After a chemokine binds to the extracellular domain of the receptor, downstream activation of PI3K leads to the activation of BTK (Fig. 1) [49]. Furthermore, the Gα and Gβγ subunits can both interact with the PH and the Tec homology (TH) domain to directly bind to BTK [50, 51]. However, the Gα subunit has been shown to directly activate BTK alone [52]. B cells express chemokine receptors such as CXCR4 and CXCR5, and BTK is critical in acting downstream to mediate chemokine-controlled migration and maintain cell homeostasis [45].

The Fc receptor is an antibody receptor expressed by a multitude of cells including B cells, macrophages, mast cells, natural killer (NK) cells, and neutrophils. These receptors recognize the constant region of immunoglobulin G, known as the Fc (fragment, crystallizable) region. Among others, the Fc epsilon receptor (FCεRI)-mediated cross-linking in mast cells strongly activates BTK, which plays a role in many Fc receptors [12, 52, 53]. An activating Fc receptor (FcεRI) and immunoreceptor tyrosine-based activation motifs (ITAMs) recruit downstream messengers to activate various kinases such as SYK and BTK upon antigen recognition and cross-linking, but if an inhibitory Fc receptor such as FcγRIIB recognizes an antigen, immunoreceptor tyrosine-based inhibitory motifs (ITIMs) recruit phosphatases to inhibit the effector function of BTK and eliminate downstream signaling (Fig. 1) [54]. Importantly, Bolland et al. reported that membrane recruitment of SHIP is responsible for the inhibitory signal generated by FcγRIIB to the BCR [55].

There is evidence that BTK can play a role in the cellular process of phagocytosis (Fig. 1). It was reported in a murine macrophage cell line RAW 264.7 that BTK was activated throughout phagocytosis and inhibition of BTK resulted in significantly inhibited FcγR-mediated phagocytosis [56]. BTK also contributes to phagocytosis in rodent microglia and human monocyte-derived macrophages as well as in cells from patients with XLA, a condition caused by a BTK mutation [57, 58]. However, further studies are needed to determine whether these effects are a result of a direct effect of the BTK inhibition.

## BTK inhibition

Due to its role in B cell development, the BCR signaling cascade, and other immune-related pathways, BTK serves as a unique therapeutic target for many B cell malignancies and other disease states. Multiple agents have been developed for BTK inhibition, [33, 59] but this review will focus on the BTK inhibitors ibrutinib and acalabrutinib which have been tested extensively in the clinical realm.

Ibrutinib or PCI-32765 (brand name Imbruvica) is an orally available, irreversible inhibitor of BTK that binds to the cysteine at position 481 in the kinase domain where it blocks BTK kinase activity [60, 61]. Ibrutinib has been evaluated in several preclinical studies since its initial discovery in 2007. Ibrutinib inhibits the autophosphorylation of BTK, the phosphorylation of its substrate PLCγ2, and the downstream extracellular-signal-regulated kinase (ERK), leading to reduced activation of NF-κB signal transduction [33, 62]. Furthermore, ibrutinib has a potent (inhibitory concentration 50 (IC_50_) = 0.5 nM) selectivity for BTK in B cells as opposed to other Tec kinases, making it an attractive therapeutic approach for B cell malignancies [33]. An important in vivo study of ibrutinib by Honiberg et al*.* reported objective clinical responses in dogs with spontaneous B cell non-Hodgkin lymphoma [55]. Furthermore, the report indicated that ibrutinib-targeted BTK yet displayed off-target effects on other kinases with a corresponding cysteine residue in the ATP-binding site including JAK and ITK. These off-target effects of ibrutinib could explain unique toxicities and why ibrutinib has been found useful in other diseases [63–68]. Clinical efficacy was first reported in patients with chronic lymphocytic leukemia (CLL) and mantle cell lymphoma (MCL) [69]. Ibrutinib received breakthrough designation and was later approved by the Food and Drug Administration (FDA) for treatment of MCL in 2013 and CLL in 2014 [20, 21].

Acalabrutinib or ACP-196 (brand name Calquence) is an orally available, more selective second-generation irreversible BTK inhibitor that was designed to improve the safety and efficacy of first-generation inhibitors like ibrutinib [70]. Acalabrutinib binds irreversibly to cysteine 481 in the BTK kinase domain and blocks kinase activity, but results in less off-target activity on kinases like EGFR and is predicted to have fewer adverse events (AEs) than ibrutinib, such as antiplatelet activity [70, 71]. Acalabrutinib has shown success in clinical trials, including the first clinical use of the BTK inhibitor for patients with relapsed CLL such as those with chromosome 17p13.1 deletion. In this clinical setting, a 95% overall response rate was reported [72]. Furthermore, a phase II trial in refractory MCL patients reported a complete response in 40% of patients [73]. Acalabrutinib became FDA approved for adult patients with MCL in 2017 and CLL in 2019. An ongoing phase III clinical study investigating the relative effectiveness of acalabrutinib and ibrutinib in previously treated subjects with CLL (NCT02477696) has pending results [74].

## The role of BTK in myeloid-derived suppressor cells (MDSC)

Myeloid-derived suppressor cells (MDSC) are a population of heterogeneous myeloid progenitor cells that can inhibit T cell function and have been identified as contributors to the progression of many different types of cancer [75, 76]. MDSC are known to expand and activate as the result of two distinct signals. The first signal functions to expand immature myeloid cell populations, while the second signal is an activation signal, primarily mediated by the transcription factor NF-κB [77]. After activation, MDSC are responsible for producing various anti-inflammatory cytokines, nitric oxide, reactive oxygen species (ROS), and arginase-1, all of which function to suppress immune function [75, 76]. BTK plays an important role in the maturation and function of myeloid cells, and targeting BTK in malignant B cells has been shown to inhibit NF-κB signal transduction [14, 15, 17, 62]. Notably, NF-κB is a demonstrated mediator of MDSC expansion and function [78, 79]. MDSC expansion can cause loss of immune effector cell function and reduce the efficacy of immune-based cancer treatments, highlighting the therapeutic benefit of potentially targeting BTK in the MDSC population [17].

Stiff et al. found that MDSC isolated from Balb/c mice bearing EMT6 and 4T1 mammary carcinoma tumors, human MDSC generated in vitro, and MDSC isolated from metastatic melanoma patients all expressed BTK [17]. Furthermore, treatment of human MDSC and the murine MDSC cell line MSC2 with the BTK inhibitor ibrutinib led to inhibition of BTK phosphorylation in these cells [17]. Notably, ibrutinib also significantly inhibited the in vitro generation of MDSC, impaired MDSC migration, reduced mRNA expression of the immunosuppressive factor indolamine 2,3-dioxygenase, and halted production of the immunosuppressive molecule nitric oxide (NO) [17]. Our group has previously shown that cancer patient MDSC produce NO which significantly inhibits NK cell Fc receptor-mediated functions, and eliminating MDSC in vivo significantly improved monoclonal antibody treatment in an EMT6–HER2 mammary carcinoma model [80]. Ibrutinib treatment in wild-type mice bearing B16F10 melanoma tumors also resulted in a significant reduction of MDSC [17]. In Balb/c mice bearing EMT6 murine mammary carcinoma tumors, treatment with ibrutinib resulted in a significant reduction of MDSC in both the spleen and tumor and was shown to significantly enhance anti-PD-L1 therapy [17]. Taken together, these results demonstrated that targeting BTK with ibrutinib can modulate MDSC function, generation, and migration, revealing a promising strategy for enhancing immune modulation for solid tumor therapies.

The ability of ibrutinib to modulate MDSC has also been demonstrated utilizing an orthotopic mouse breast cancer model [81]. Varikuti et al. reported that ibrutinib treatment inhibited the proliferation of the E0.2 breast cancer cell line in vitro, while additionally demonstrating through a proliferation assay with CFSE-treated cells (carboxyfluorescein succinimidyl ester) that T cells co-cultured with MDSC from the spleens of ibrutinib-treated mice can proliferate significantly more compared to controls while promoting effector functions leading to the induction of anti-tumor T helper type 1 (Th1) and cytotoxic T lymphocytes (CTL) immune responses [17, 81]. Additionally, the spleens and tumors of ibrutinib-treated mice had significantly reduced levels of monocytic MDSC (M-MDSC), but the levels of granulocytic MDSC (PMN-MDSC) exhibited no significant change [81]. High levels of M-MDSC have been correlated with breast cancer metastasis and progression in human breast cancer patients [82]. Thus, it was not surprising for this group to find that ibrutinib-treated mice had a significant reduction in tumor metastasis to the lungs, a lower tumor burden, and reduced breast tumor progression compared to controls [81]. Treatment with ibrutinib also increased the frequency of mature dendritic cells (DCs) in the spleens and tumors of mice by converting MDSC to mature DCs, which are known to play a critical role in anti-tumor immunity [81–83]. These findings were confirmed via an ex vivo treatment of MDSC with ibrutinib which significantly enhanced expression of CD11c and MHCII molecules, indicating a change in phenotype to mature DCs [81]. These findings suggest the BTK pathway negatively regulates the conversion of MDSC into mature DCs [81]. Overall, these results demonstrate that ibrutinib inhibits tumor development and metastasis in an E0.2 breast cancer mouse model by inducing the transformation of mature DCs from MDSC, providing a novel, promising approach for the treatment of human breast cancer [81].

It is hypothesized that MDSC could limit the effectiveness of checkpoint inhibitors and that BTK inhibition could improve the effectiveness of checkpoint inhibitors by eliminating the negative effects of MDSC. As a result, combination clinical studies are being performed with BTK and checkpoint inhibition in patients with solid malignancies (Table 1). Overman et al. recently conducted a phase II, multicenter, open-label, randomized (1:1) clinical trial (NCT02362048) evaluating the BTK inhibitor acalabrutinib alone or in combination with the anti-PD-1 antibody pembrolizumab in patients with advanced pancreatic cancer [84]. Overall, 77 patients (37 monotherapy, 40 combination therapy) with a median age of 64 years received 100 mg of oral acalabrutinib twice daily with (combination therapy) or without (monotherapy) 200 mg of intravenous pembrolizumab on day 1 of each 3-week cycle, as peripheral blood and some tumors were analyzed [84]. The elevation of peripheral MDSC has been correlated with disease progression in patients with solid tumors treated with anti-cytotoxic T-lymphocyte-associated protein-4 (anti-CTLA-4), anti-PD-1, or anti-PD-L1 therapies [85, 86]. Flow cytometric analysis of peripheral blood mononuclear cells (PBMC) demonstrated durable reductions in the levels of PMN-MDSC, with a median reduction of greater than 50% achieved after 2–3 weeks of therapy in both arms of the study, suggesting this effect was likely facilitated by the BTK inhibitor acalabrutinib [84]. Furthermore, two exceptional responders in the study were shown to have low tumor mutational burden and no defects in the homologous DNA repair pathway [84]. Overall, the Overman et al. study showed that combination therapy with ibrutinib and pembrolizumab was well tolerated in patients with advanced pancreatic cancer, but limited clinical benefit was achieved in both arms of the study [84]. However, the study did demonstrate the strong effect on eliminating peripheral PMN-MDSC levels, emphasizing the importance of further studying the role of MDSC in the tumor microenvironment. Furthermore, a phase I clinical trial (NCT03525925), studying the effect of ibrutinib and anti-PD-1 inhibitor nivolumab on the levels of circulating MDSC in patients with metastatic solid tumors, is ongoing at our institution. Early results from this study show an initial increase in circulating MDSC levels with single-agent ibrutinib followed by reductions with the combination regimen. Plasma levels of chemokines (IL-8, CCL2, CCL3, and CCL4) associated with MDSC recruitment and migration significantly decreased following ibrutinib treatment and T cell function significantly improved with the combination therapy. Taken together, these results demonstrate the ability of ibrutinib to modulate MDSC in the setting of cancer and provide biological evidence to support the expansion of strategies to target MDSC in combination with immune checkpoint inhibition [87]. Overall, BTK inhibition has shown a favorable benefit in solid tumors with anti-tumor activity; however, these data are still preliminary, and it is possible that these effects are a result of the off-target effects of BTK inhibitors on other kinases.

**Table 1:** An overview of BTK and checkpoint inhibition combination clinical trials in solid malignancies

Combination		Setting	Phase	NCI identifier	Recruitment status	Effect
BTK inhibitor	Checkpoint inhibitor					
Ibrutinib	Pembrolizumab	Advanced refractory colorectal cancers	I/II	NCT03332498	Active, not recruiting	
Ibrutinib	Pembrolizumab	Stage III–IV melanoma that cannot be removed by surgery	II	NCT03021460	Recruiting	
Ibrutinib	Pembrolizumab	Gastrointestinal and genitourinary tumors	I/II	NCT02599324	Recruiting	
Ibrutinib	Nivolumab	Metastatic solid tumors	I	NCT03525925	Active, not recruiting	
Ibrutinib	Nivolumab	Non-small cell lung cancer	II	NCT02950038	Withdrawn	
Ibrutinib	Nivolumab	Previously treated metastatic kidney cancer	I/II	NCT02899078	Recruiting	
Ibrutinib	Nivolumab	Recurrent/metastatic HNSCC	II	NCT03646461	Recruiting	
Ibrutinib	Durvalumab	Relapsed or refractory solid tumors	I/II	NCT02403271	Completed	122 patients were enrolled and the combination had an acceptable safety profile. Overall response rates (complete or partial responses) were 2% for pancreatic cancer, 3% for breast cancer, and 0% for NSCLC
Acalabrutinib	Pembrolizumab	Advanced or metastatic pancreatic cancer	II	NCT02362048	Completed	77 patients were enrolled (37 monotherapy; 40 combination therapy) and the combination was well tolerated. The overall response rate was 0% with monotherapy and 7.9% with combination therapy
Acalabrutinib	Pembrolizumab	Advanced non-small cell lung cancer	II	NCT02448303	Completed	31 patients and 28 patients were enrolled in the monotherapy and combination therapies, respectively, with overall response rates of 12.9% and 14.3%
Acalabrutinib	Pembrolizumab	Advanced head and neck squamous cell carcinoma	II	NCT02454179	Completed	37 patients and 30 patients were enrolled in the monotherapy and combination therapies, respectively, with overall response rates of 18.9% and 16.7%
Acalabrutinib	Pembrolizumab	Ovarian cancer	II	NCT02537444	Completed	35 patients and 33 patients were enrolled in the monotherapy and combination therapies, respectively, with overall response rates of 2.9% and 9.1%
Acalabrutinib	Pembrolizumab	Metastatic urothelial carcinoma	II	NCT02351739	Completed	31 patients and 34 patients were enrolled in the monotherapy and combination therapies, respectively, with overall response rates of 29% and 23.5%

## The role of BTK in tumor-associated macrophages (TAM)

In cancer, macrophages can be recruited to the tumor microenvironment, and their function modified by tumor-derived factors. These tumor-resident macrophages may be roughly characterized as tumor-associated macrophages (TAM) [88]. Importantly, TAM can promote tumor growth and metastasis, enhance cancer cell resistance to chemotherapy, activate immunosuppression pathways, and inactivate T cell effector functions [88, 89]. Sousa et al. demonstrated in a study of human breast cancer that higher levels of TAM were correlated with increased tumor cell proliferation, augmented tumor growth, significant immunosuppression, and a subsequent increase in disease burden [90]. TAM are one of the most abundant tumor-infiltrating immune cells in the tumor microenvironment and play an important role in suppressing the anticancer immune response. Thus, TAM have become an attractive therapeutic target [91].

One mechanism by which TAM protect cancer cells and promote pro-survival signaling is through the secretion of chemokines such as CXCL12 and CXCL13, which bind to the G-protein-coupled receptors CXCR4 and CXCR5, respectively, found on malignant cells [92]. Due to the role that BTK plays in macrophage production of many pro-inflammatory chemokines and cytokines, Ping et al. investigated the immunomodulatory effects of BTK inhibition on macrophages [92]. This group discovered that BTK inhibition with ibrutinib or with PLS-123 (targets BTK catalytic activity and its auto-activation) in LPS-stimulated THP-1 differentiated macrophages efficiently downregulated the secretion of the homeostatic chemokines CXCL12, CXCL13, and CCL19, as well as the angiogenic cytokine VEGF [92]. Inhibition of these factors also occurred at the mRNA level, demonstrating the role of BTK in regulating the transcription of these genes [92]. In addition to targeting BTK pharmacologically, Ping et al. performed siRNA-mediated knockdown of BTK which also significantly impaired the secretion of CXCL12, CXCL13, CCL19, and VEGF [92]. Moreover, macrophages co-cultured with malignant B cell lymphoma cells (Namalwa or OCI-Ly7 cell lines) exhibited diminished adhesion to fibronectin compared to control [92]. A transwell migration assay demonstrated that malignant B cell and T cell lymphoma cells had diminished ability to migrate and invade when cultured with the supernatants of ibrutinib-treated cells [92]. Further experiments in macrophages revealed that BTK inhibitors impede downstream signaling pathways such as PLCγ2 and MAP kinases and the activity of specific transcription factors important for controlling chemokine and cytokine production such as NF-κB, STAT3, and AP-1 [92]. These results suggest that BTK inhibitors can be used to target TAM in the tumor microenvironment and modify the immune landscape via modulation of the chemokine and cytokine milieu.

Others have studied the role of TAM in murine models of cancer, particularly in pancreatic ductal adenocarcinoma (PDAC)-bearing mice [93]. One characteristic feature of PDAC is the infiltration of various lymphoid and myeloid cell lineages, like TAM, which exert a strong effect on the tumor microenvironment and often contribute toward the ineffectiveness of various therapies [94]. Gunderson et al. investigated the role of the BTK signaling pathway based on data indicating that B cells and TAM can contribute to PDAC disease progression and tumorigenesis by inhibiting cytotoxic T lymphocyte (CTL) responses. This group found that PDAC tumor growth requires crosstalk between B cells and TAM, resulting in pro-tumor Th2-type macrophage polarization via activation of the BTK signaling cascade [93]. In fact, one of the main features of an immunosuppressive environment was the lack of functional CTL due to macrophage polarization into a Th2-type phenotype [2]. Treatment of PDAC-bearing mice with ibrutinib reprogrammed macrophages toward a Th1-type macrophage phenotype which led to the enhancement of CD8^+^ CTL responses, improvement of a chemotherapeutic intervention with gemcitabine, and suppression of PDAC tumor progression. Thus, BTK appears to serve as a key regulator of TAM crosstalk and anti-tumor immune responses [93]. Unfortunately, in some cases in vivo ibrutinib is rapidly cleared, and thus, the low accumulation of ibrutinib results in the tumor, rendering these targeted therapies ineffective [95]. Qiu et al. have designed, synthesized, and investigated a sialic acid–stearic acid (SA) conjugate that encapsulates the BTK inhibitor ibrutinib within amphiphilic egg phosphatidylglycerol (EPG) as a novel immunotherapeutic approach to allow for prolonged exposure of TAM to ibrutinib [95]. Immunofluorescence staining demonstrated that the SA/IBR/EPG nanocomplex accumulated in TAM both in vitro and in vivo. This agent inhibited angiogenesis, Th2 tumorigenic cytokine secretion, and tumor progression and represents a promising targeted approach for targeting BTK in TAM [95].

Benner et al. have also studied the role that the BTK signaling pathway may have in modulating TAM-produced inflammatory processes [15]. The NLRP3 inflammasome is a multi-protein complex involved in innate immunity. It is composed of NLRP3, the adaptor protein ASC, and caspase-1 and is responsible for the production of the potent inflammatory cytokine IL-1β [96]. Due to the inherent role of BTK in myeloid cell signal transduction, it was hypothesized that in vitro generated TAM would express BTK and that this enzyme would participate in NLRP3 inflammasome activity [15, 97]. Murine and human TAM (both in vitro generated and tumor-derived) were utilized to establish that TAM express both BTK and the NLRP3 inflammasome [15]. Moreover, it was demonstrated that BTK physically associates with the NLRP3 inflammasome and promotes its activation and subsequent ability to mediate the production of IL-1β [15]. IL-1β production could be induced in TAM using LPS and ATP co-stimulation and ibrutinib inhibited the phosphorylation of BTK, prevented the association of BTK with the NLRP3 inflammasome, and reduced inflammasome activation as evidenced by a decrease in IL-1β production [15]. Furthermore, compared to controls, systemic ibrutinib therapy led to reduced expression of IL-1β expression in murine EMT6 and 4T1 tumors. These results suggest that BTK plays an important role in TAM-mediated inflammation within the tumor microenvironment that involves around IL-1β [15].

Other groups have studied the association between BTK and the NLRP3 inflammasome in different disease settings. Liu et al. used proteome-wide phosphoproteomics to identify and study BTK as a novel regulator of the NLRP3 inflammasome [18]. Utilizing bone marrow-derived macrophages from BTK-deficient mice and PBMC from patients with X-linked agammaglobulinemia (XLA), this group found reduced IL-1β production and impaired inflammasome activation within mutant myeloid cells [18]*.* Also, BTK inhibition impaired IL-1β processing and release in human primary macrophages from healthy donors, ibrutinib-treated cancer patients, and patients with Muckle–Wells syndrome—an autoinflammatory disease characterized by excessive production of IL-1β [18]. Inflammasome activation has also been implicated in the post-ischemic inflammation that occurs after stroke [98, 99]. Using a murine model of brain ischemia/reperfusion, Ito et al. reported that BTK inhibition by genetic and pharmacologic means impaired inflammasome activation in brain infarct macrophages and exerted a neuroprotective effect [14]. Immunofluorescence staining demonstrated that BTK inhibition suppressed caspase-1 activation in the brain infarct and correlated with the reduced maturation of IL-1β [14]. These studies offer further evidence for BTK as a crucial mediator of myeloid cell function and NLRP3 inflammasome activation and point toward the potential for BTK inhibition as a means of improving clinical outcomes in cancer and other diseases.

## Targeting BTK beyond the tumor microenvironment

Myeloid cell lineages contribute to inflammation and infection, making BTK an interesting candidate for therapeutic intervention in a variety of disease settings [100]. TLR signaling plays an important role in innate immunity and TLRs are expressed in many myeloid cell populations including dendritic cells, mast cells, and macrophages, which are all essential for the recognition and elimination of microbial pathogens [101]. BTK promotes TLR signaling, leading to the activation of pro-inflammatory cytokines resulting in robust immune responses in many bacterial infections like pneumococcal pneumonia [102]. Murine models have demonstrated the protection BTK confers from endotoxin shock caused by the Gram-negative bacteria *Escherichia coli* [40]. Furthermore, BTK regulates macrophage responses to the Gram-positive bacteria *Listeria monocytogenes* and *Staphylococcus aureus* infections [103, 104]. In filarial infections, Mukhopadhyay et al*.* revealed that BTK-mutant CBA/N mice showed delayed clearance of microfilaria compared to wild-type CBA/N mice and that BTK regulates macrophage effector functions such as bactericidal activity and secretion of pro-inflammatory cytokines [10, 105]. However, the BTK inhibitor ibrutinib has been shown to impair immune mechanisms dedicated to controlling the *Mycobacterium tuberculosis* infection [106].

BTK is also involved in the clearance of fungal infections. Specifically, BTK contributes to the dectin-1-dependent phagocytosis of *Candida albicans* by macrophages [107]. Furthermore, Shahan et al*.* demonstrated that BTK is activated in the presence of spores from the fungal species *Aspergillus niger*, *Aspergillus candidus, and Eurotium amstelodami* [108]*.* BTK-deficient XID mice had enhanced susceptibility to *Cryptococcus neoformans* infection*,* a fungal pathogen that can affect immunocompromised patients [109]. In other studies, inhibition of the BTK signaling pathway with ibrutinib led to reduced NF-κB signaling and resulted in an increased risk of developing *Aspergillus fumigatus*, an invasive fungus largely found in organ transplant recipients [110, 111]. Recently, others have also demonstrated that second-generation BTK inhibitors impair the antifungal response of macrophages [112].

Various inflammatory diseases are implicated with the BTK signaling pathway. BTK regulates osteoclast differentiation by linking receptor activator of nuclear factor-κB (RANK) and immunoreceptor tyrosine-based activation motif (ITAM) signals, and inhibiting BTK protects against osteoclast-mediated bone loss disorders [16, 113]. Other groups have shown that BTK is needed to drive macrophage activation and could be a potential target for patients with rheumatoid arthritis and sepsis [114, 115]. BTK inhibition ameliorates autoimmune arthritis and treatsTLR7/IFN-driven murine lupus, while BTK-deficient XID mice show reduced severity in many inflammatory disease models such as autoimmune encephalomyelitis (EAE), colitis, and acute edema [97, 116, 117]. Finally, Mao et al*.* demonstrated that BTK regulates NLRP3 inflammasome activity in the setting of Crohn’s disease [118].

In addition to inflammatory diseases, bacterial and fungal infections, BTK plays a critical role in initiating antiviral responses [119]. In some pulmonary viral infections, the macrophage response driven by BTK can lead to tissue damage and unfavorable outcomes. For example, in murine models of influenza viral infection BTK inhibition with ibrutinib decreased lung injury and led to reduced alveolar macrophage activation [120]. Recently, Chong et al*.* and others discussed the potential that BTK inhibition may have in treating the novel virus SARS-CoV-2 (COVID-19) [121–123]. Pulmonary failure is the main cause of mortality related to COVID-19 infection, and BTK inhibition may mitigate the hyper-inflammatory pulmonary response through attenuation of M1-macrophage polarization [121, 124, 125]. Our group presented that the BTK inhibitor ibrutinib could inhibit inflammasome activation in TAMs and reduce the release of IL-1β (see below) [15]. These findings suggest a potential mechanism by which ibrutinib can inhibit macrophage activation, lower the production of IL-1β, and alter the pulmonary inflammatory landscape in patients infected with COVID-19. Furthermore, one clinical study utilized the BTK inhibitor acalabrutinib in COVID-19 patients. Administration of acalabrutinib led to improved oxygenation in a majority of patients over the 10–14-day treatment course and was well tolerated. Measures of inflammation such as C-reactive protein and IL-6 rapidly returned to normal in most patients, as did lymphopenia [122]. These findings support the hypothesis that the release of pro-inflammatory cytokines by pulmonary macrophages in COVID-19 is a major contributor to pulmonary failure and that BTK inhibition could provide some degree of protection against lung injury in this setting. BTK plays a large role in macrophage polarization, and through a variety of transcription factors, it may effectively regulate the hyperinflammatory state associated with many microbial infections and inflammatory disease settings.

## Conclusion

Much progress has been made in understanding the mechanism by which BTK regulates innate immunity. Targeting BTK in CLL could have significant clinical efficacy via inhibition of the BCR signaling pathway in addition to the significant effects of BTK inhibition on B cell adhesion and chemotaxis. Moreover, targeting BTK in myeloid cells has shown recent progress and holds therapeutic promise for modulating the tumor microenvironment. However, more work is needed to determine the clinical efficacy of BTK inhibition in various disease states. One avenue of particular interest is utilizing BTK inhibition in combination with other immune-modulating agents to combat resistance to single-agent therapies. A greater understanding of the differences in safety profiles and clinical benefit of available BTK inhibitors in different disease states is needed in order to design effective treatment strategies.
